# Supplementary material for: Netcombin: An algorithm for constructing optimal phylogenetic network from rooted triplets
Source: PLoS One. 2020 Sep 18;15(9):e0227842. doi: 10.1371/journal.pone.0227842 (PMC7500971; doi:10.1371/journal.pone.0227842)
Supplement: S4 File — (ZIP) [file pone.0227842.s004.zip › SequenceData-Triplet/sequenceData-10-20-30-0-Triplet/length 300, numseq 30, rate 10^-7/4 seq.pdf]

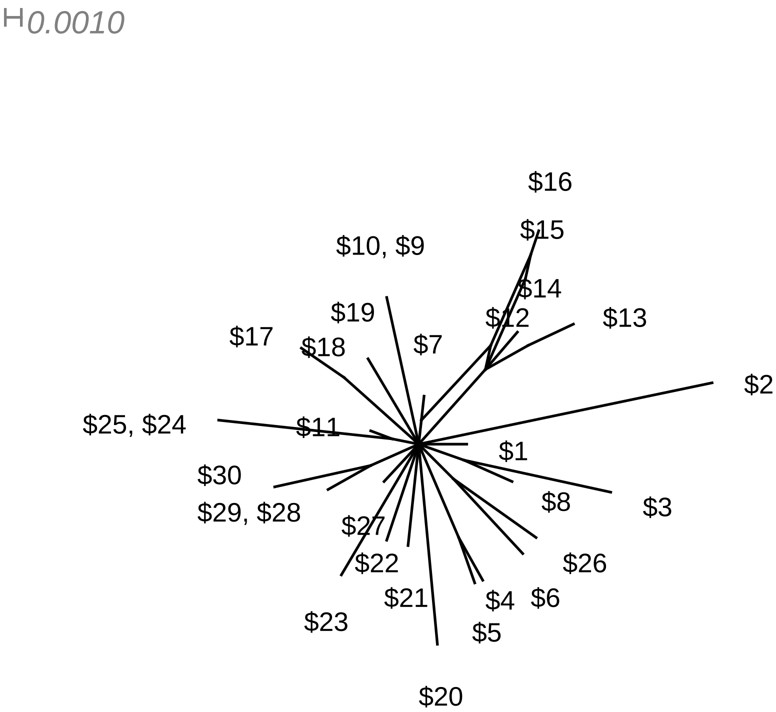
0.0010
$16
$15
$10, $9
$14
$19
$12
$13
$17
$18
$7
$2
$25, $24
$11
$1
$30
$8
$3
$29, $28
$27
$26
$22
$21
$4
$6
$23
$5
$20
